# Spillover of West Caucasian Bat Lyssavirus (WCBV) in a Domestic Cat and Westward Expansion in the Palearctic Region

**DOI:** 10.3390/v13102064

**Published:** 2021-10-14

**Authors:** Stefania Leopardi, Ettore Barneschi, Giuseppe Manna, Barbara Zecchin, Pamela Priori, Petra Drzewnioková, Francesca Festa, Andrea Lombardo, Fabio Parca, Dino Scaravelli, Andrea Maroni Ponti, Paola De Benedictis

**Affiliations:** 1 National (Italy) and FAO Reference Center for Rabies, Istituto Zooprofilattico Sperimentale delle Venezie, 35020 Legnaro, PD, Italy; bazecchin@izsvenezie.it (B.Z.); pdrzewniokova@izsvenezie.it (P.D.); ffesta@izsvenezie.it (F.F.); pdebenedictis@izsvenezie.it (P.D.B.); 2 Area Sanità Animale, AUSL Toscana SudEst, 52100 Arezzo, AR, Italy; ettore.barneschi@uslsudest.toscana.it (E.B.); fabio.parca@uslsudest.toscana.it (F.P.); 3 Virology Laboratory, Istituto Zooprofilattico Sperimentale del Lazio e della Toscana, 00178 Rome, RM, Italy; giuseppe.manna@izslt.it; 4 Studi Ecologici Ricerca Natura Ambiente, 47121 Forlì, FC, Italy; pamela.priori@gmail.com (P.P.); dino.scaravelli@gmail.com (D.S.); 5 Local Department of Central Tuscany, Istituto Zooprofilattico Sperimentale del Lazio e della Toscana, 52100 Arezzo, AR, Italy; andrea.lombardo@izslt.it; 6 Direzione Generale della Sanità Animale e dei Farmaci Veterinari, Italian Ministry of Health, 00153 Rome, RM, Italy; a.maroni@sanita.it

**Keywords:** West Caucasian Bat Lyssavirus, animal–human encroachment, bats, *Miniopterus schreibersii*, spillover

## Abstract

In June 2020, a cat from Arezzo (Italy) that died from a neurological disease was diagnosed with West Caucasian Bat Lyssavirus (WCBV). The virus retained high identity across the whole-genome with the reference isolate found in 2002 from a Russian bent-winged bat. We applied control measures recommended by national regulations, investigated a possible interface between cats and bats using visual inspections, bioacoustics analyses and camera trapping and performed active and passive surveillance in bats to trace the source of infection. People that were exposed to the cat received full post-exposure prophylaxis while animals underwent six months of quarantine. One year later, they are all healthy. In a tunnel located near the cat’s house, we identified a group of bent-winged bats that showed virus-neutralizing antibodies to WCBV across four sampling occasions, but no virus in salivary swabs. Carcasses from other bat species were all negative. This description of WCBV in a non-flying mammal confirms that this virus can cause clinical rabies in the absence of preventive and therapeutic measures, and highlights the lack of international guidelines against divergent lyssaviruses. We detected bent-winged bats as the most probable source of infection, testifying the encroachment between these bats and pets/human in urban areas and confirming free-ranging cats as potential hazard for public health and conservation.

## 1. Introduction

Rabies is a major zoonosis caused by viruses of the genus *Lyssavirus. Rabies Virus* (RABV) is responsible for the vast majority of rabies cases worldwide, but all lyssaviruses cause the same indistinguishable clinical presentation and outcome [1]. Currently, the International Committee on Taxonomy of Viruses recognizes 17 viral species in this genus, with another three awaiting official classification [2,3]. All known lyssaviruses but two have been associated with species of the order *Chiroptera* [4,5]. Indeed, different bats are infected with lyssaviruses worldwide [4,6,7]. This includes RABV only in the Americas, where multiple viral variants circulate among insectivorous, frugivorous and hematophagous bat species and are responsible for spillover events to other wildlife, livestock, pets and humans [8,9,10,11,12]. Field and experimental data agree that cross-species transmission of lyssaviruses mostly results in dead-end infections, with no perpetuation in the new host [6,7,13,14,15,16,17]. Interestingly, each RABV variant and lyssavirus species found in bats seems to be associated with one or few related species, suggesting an evolutionary adaptation to the native host [17].

Lyssaviruses are classified into three phylogroups (I, II and III) based on genetic and antigenic differences [1]. The most divergent is phylogroup III that includes four viruses, namely *West Caucasian Bat Lyssavirus* (WCBV), *Lleida bat Lyssavirus* (LLEBV), *Ikoma lyssavirus* and the newly described *Matlo bat lyssavirus* (MBLV) [3,18,19]. Among these, WCBV and LLEBV have been described in the Schreibers’ bent-winged bat (*Miniopterus schreibersii*) and MBLV was discovered in South Africa from *Miniopterus natalensis,* suggesting that the genus *Miniopterus* might have a major role in the evolution of divergent lyssaviruses [3]. Critically, current vaccine formulations are all based on RABV and are known to cover only for viruses belonging to phylogroup I [2,20,21,22].

Carnivore rabies, including fox rabies, is mostly under control in the European Union (EU), except for the recent re-emergence of the disease in Poland and Romania [23,24,25]. However, five lyssaviruses are reported to circulate throughout the EU in bats of the genera *Myotis, Eptesicus* and *Miniopterus.* Among these, *European bat 1 lyssavirus* (EBLV-1), *European bat 2 lyssavirus* (EBLV-2) and *Bokeloh bat lyssavirus* are frequently reported, while *Lleida bat lyssavirus* and *Kotalahti bat lyssavirus* are only sporadically described [5,26]. In addition, antibodies neutralizing some of these lyssaviruses have been found in several other species, mostly referring to EBLV-1 and EBLV-2, suggesting that either the diversity or the host range of these viruses is underestimated. As an example, at least three non-reservoir bat species, namely *Myotis myotis, Myotis blythii* and *Tadarida teniotis*, have been found to react against EBLV-1 in Italy, despite no positive cases confirmed to date [27].

On 26 June 2020, the Virology Unit of the Istituto Zooprofilattico Sperimentale del Lazio e della Toscana (IZSLT) based in Rome, one of the seven regional laboratories enabled to diagnose animal rabies in Italy and responsible for the Tuscan territory, notified a rabies infection in a domestic cat living in Arezzo, Tuscany, central Italy. On 27 June the case was confirmed by the National Reference Centre for Rabies at the Istituto Zooprofilattico Sperimentale delle Venezie (IZSVe), which identified WCBV as the etiological agent, a virus previously reported only once in 2002 from an healthy Schreibers’ bent-winged bat that was euthanized in the Caucasus Mountains (Russia) during active rabies surveillance [19]. Here we report the control measures implemented during the outbreak and the investigations that followed the diagnosis carried out with the aim of tracing the source and the triggers for the viral spillover, which encompassed viral, epidemiological and ecological investigations.

## 2. Materials and Methods

### 2.1. Outbreak Investigation and Control Measures

Upon diagnosis, the Italian Ministry of Health convened a task force to track and manage the cases of exposure and to prevent the occurrence of secondary cases. The group was made up of members from the Veterinary and Prevention Departments of the Ministry of Health, the Italian Institute for Environmental Protection and Research, the Tuscany Region, the major of Arezzo, the Public Veterinary Services of the Arezzo province, and the laboratories involved, namely IZSVe and IZSLT.

To trace all the possible contacts the infected cat may have had with people and animals up to 10 days before the onset of disease, we organized detailed interviews with the owners and the veterinarians employed in the two clinics where the cat had been assessed and hospitalized. We investigated the cat’s habits, its anamnesis, the progression of the disease and the occurrence of bites and scratches. All the people that had likely to have been in contact with the cat received full post exposure prophylaxis (PEP), including the administration of rabies immunoglobulins (RIG), and have been kept under medical surveillance since then.

In the urban area of Arezzo we implemented all the control measures recommended by the national regulation (DPR 320, 1954) in case of confirmed rabies cases [28]. These include: (i) a six-month quarantine in a public shelter under veterinary surveillance for all domestic animals exposed or presumably exposed to the infected cat, (ii) a muzzle and leash obligation for all dogs for a period of two months following the diagnosis, and (iii) the obligation for all pet owners to immediately report any aberrant behaviour or neurological signs of their animal or if their pets were missing. To facilitate communications between the public health sector and the citizen, the municipality of Arezzo set a 24 h call centre and promoted an informative campaign about the risks related to WCBV, particularly in the index neighbourhood. In addition, all campaigns for the sterilization of stray cats were suspended for two months to secure the safety of veterinarians. On the other hand, stray cats were subject to longitudinal surveillance to monitor the onset of clinical signs or any suspected behaviour, which included regular inspections performed by veterinarians in 13 colonies located within a radius of 3 km from the cats’ house and several interviews with the personnel responsible for these facilities. In detail, cat colonies are locations where strayed cats find appropriate sheltering and are fed. These aggregations are licensed and managed under strict sanitary regulations by the municipality of Arezzo [29].

Considering that the clinical disease associated with WCBV was indistinguishable from that caused by RABV and according to the OIE Terrestrial Animal Health Code, we defined as a suspect case “any susceptible animal that shows a change in behaviour followed by death within ten days or that displays any of the following clinical signs: hyper-salivation, paralysis, lethargy, abnormal aggression, abnormal vocalisation” [30]. All suspect cases were submitted to IZSLT to rule out rabies [30].

### 2.2. Spillover Investigation

Considering that Italy is free of rabies infection (caused by RABV) [31,32] and that the cat had no history of travelling abroad, and based on current knowledge on the ecology of lyssaviruses, we assumed the animal had likely acquired the infection from an infected bat. No information was available for the occurrence of non-RABV lyssaviruses in the area. Thus, we enhanced passive surveillance in bats by a high-throughput communication with wildlife rehabilitation centres, bat handlers and local authorities and we screened all available carcasses regardless of the species, sex, age and presence of clinical disease.

We investigated where, when and how the cat may have come in contact with bats and to do this we used visual inspections, bioacoustics analyses and camera trapping. We determined the most likely home-range of the cat based on its sex, reproductive status and habits [33] and we screened the area for bat roosts in ruins, tunnels and tree cavities, also using an endoscopic camera. We paid particular attention to the underground stretch of a river that runs below the city for about two kilometres and whose access is located in front of the cat’s house. The whole length of this site was inspected monthly, leading to the discovery, in the first third of August, of a group of *Miniopterus schreibersii scheribersii* that, up to now, is acknowledged as the only confirmed natural host of WCBV. To describe the presence and habits of both bats and cats in this site that could somehow have favoured the spillover, we positioned two automatic fixed bat detectors (D500X, Petterson Elettronik AB, Uppsala, Sweden) on the two entrances of the tunnel and a camera trap in the index side only, where the river is dry during most of the year. Recordings were analyzed with BatSound 4.12 (Pettersson Elektronik AB, Uppsala, Sweden) and Raven Pro 1.5 (Cornell lab of ornithology, Ithaca, NY, USA) as described elsewhere [34]. Briefly, all the ultrasounds referred to bats were first analysed to determine the bat species [35] and then classified in echolocations calls, feeding buzzes and social calls [36]. These data were used to determine the species richness in the area and to describe in particular the activity of bent-winged bats. For example, we used the buzz-ratio, defined as the percentage of feeding buzzes over the total passes per recording night, to test if these animals spend time in the site to forage, which should increase chances for their interaction with cats. In autumn, we suspended the survey after three weeks in which no animals were either seen or recorded. As the bent-winged bat is known to sometime use the same spot in autumn and spring moving between the hibernation and the reproductive sites, we continued inspections throughout winter and re-installed back bat detectors from the beginning of March, when we also increased the frequency of our inspections. Bio-acoustic analyses were performed in spring following the same approach of late summer until the new departure of the animals in the month of June.

Bent-wing bats inhabiting the underground river were sampled to investigate the circulation of WCBV four times within one year, twice in late summer-autumn and twice in spring, capturing as many individuals as possible during the day using hand nets, for a total of 75, 82, 64 and 47 in September, October, April and May, respectively. We inspected all individuals to confirm the species and to determine their age, sex and physiological status [37]. We collected 20–90 µL of blood from the interfemoral vein in the uropatagium using a 300 µL insulin syringe with a 30 G needle, and a salivary sample using sterile nylon paediatric tracheal swabs (COPAN, Brescia, Italy). We performed all procedures under physical restraint for a maximum of 6 min/individual; after sampling, we provided some water to drink using a 1 mL syringe without a needle to prevent dehydration before releasing them back to the tunnel.

During active surveillance, we collected two biological samples from bent-winged bats, namely salivary swabs for molecular analyses aiming at virus detection and blood samples for serology. Salivary swabs were immediately placed in lysis buffer (RA1, Macherey Nagel, Düren, Germany), frozen in the field in liquid nitrogen and then stored at −80 °C until analysis.

Blood samples were stored at room temperature for a maximum of 24 h then placed at +4 °C. Once in the lab, they were centrifuged at 2700 RCF for four minutes to separate the serum that was stored at −20 °C until analysis.

### 2.3. Laboratory Analysis

Diagnosis of the index case was performed at the IZSLT through the fluorescence antibody test (DFA) and a pan-lyssavirus rRT-PCR protocol according to current OIE recommendations for animal testing. The test was performed only on brain tissue but not in salivary glands because the carcass was immediately destroyed and could not be reclaimed upon diagnosis. Typing was performed at the IZSVe through RT-PCR and sequencing of the N gene hypervariable portion (approximately 600 bp) [38,39,40] and subsequently through whole genome sequencing using a metagenomics approach implemented in the Illumina platform [41]. We assembled the genome using as a reference the sole other sequence available for WCBV (Accession number EF614258), and determined the 3′ terminal sequence as described by Marston et al. (2007) [42]. We aligned our sequences with strains of all lyssavirus species using Mafft with the G-LINS-1 parameters and other default settings [43], and performed a Maxim Likelihood phylogenetic analysis with 1000 bootstraps, using PhyML implemented in Seaview (Lyon, France) [44].

For passive surveillance, we followed a slightly different approach according to the target species. In particular, we used the sole molecular approach for bats [45], while samples from domestic animals were analysed also using DFA. The use of molecular techniques as screening method has recently been approved for the diagnosis of rabies [38]; this method provides the best performances in case of poor or scarce material, as often happens with bats. All bat carcasses were further investigated to confirm the host species through the partial sequencing of the cytochrome oxidase I, using degenerated primers [46] and relying on BOLD system as reference database [47].

After 15 s of vortexing, RNA was extracted from salivary swabs using the NucleoSpin RNA kit (Macherey-Nagel, Düren, Germany). To obtain high sensitivity for WCBV while retaining ability to detect all lyssaviruses, we tested all samples in parallel using two real-time RT-PCRs, namely LN34, which also includes the analysis of host β-actin as housekeeping gene [48], and a novel protocol where primers of LN34 were modified for an optimal match with WCBV. The performances of these tests were compared with our screening RT-PCR [40] using WCBV RNA transcribed in vitro, as detailed in the supplemental material (Figure S1).

Sera were screened for the presence of virus-neutralizing antibodies to WCBV using the Rapid Fluorescent Foci Inhibition Test (RFFIT) modified as described elsewhere [27], using as challenge virus WCBV_Arezzo_Italy_cat_2020. Briefly, the virus was isolated from the cat’s brain under BSL-3 conditions using murine neuroblastoma cells (Neuro-2a) (ATTC, Manassas, VA, USA) and was further adapted and titrated using BSR cells, a clone of baby hamster kidney cells (BHK-21) commonly used for rabies diagnosis [49]. For the analysis, serum samples were diluted 1:10 in culture medium and analyzed on a three-fold dilution basis using BSR cells; titers were calculated through the Reed-Muench method and expressed as LogD50/mL [50]. We considered as seropositive the sera that inhibited viral growth at 50% at the starting dilution, corresponding to LogD50/mL ≥ 1.48. We used reference sera against EBLV-1, EBLV-2 and RABV together with the negative control and we further screened 30 bat samples using EBLV-1 and LLEBV as challenge viruses to test for cross-reactions. We selected the sera with the highest volumes (20–40 µL) that would allow crosschecking. Serological data were analysed using the exact Fisher test implemented in Prism 6 to investigate the sex, the age of the animals and the likely influence of the month of sampling.

## 3. Results

### 3.1. Case Description and Viral Characterization

The infected cat was a neutered 2-year-old female living in town allowed to free-range outside. The owner confirmed that she was in the habit of returning home to eat, rest and sleep. She had no history of travelling abroad and no lesions consistent with fighting were reported by vet clinicians upon admission. She developed the first neurological signs on 12 June, namely aggressive behaviour, tremor, fever, abdominal pain, dysphagia, strangury and straining to defecate. The cat was clinically investigated in two independent veterinary clinics and humanely euthanized on 17 June due to the deterioration of her overall conditions and the lack of etiological diagnosis. Rabies testing was requested by the local veterinary services in compliance with the national legislation in force in Italy in case of biting and animals with neurological signs [28].

**Figure 1:**
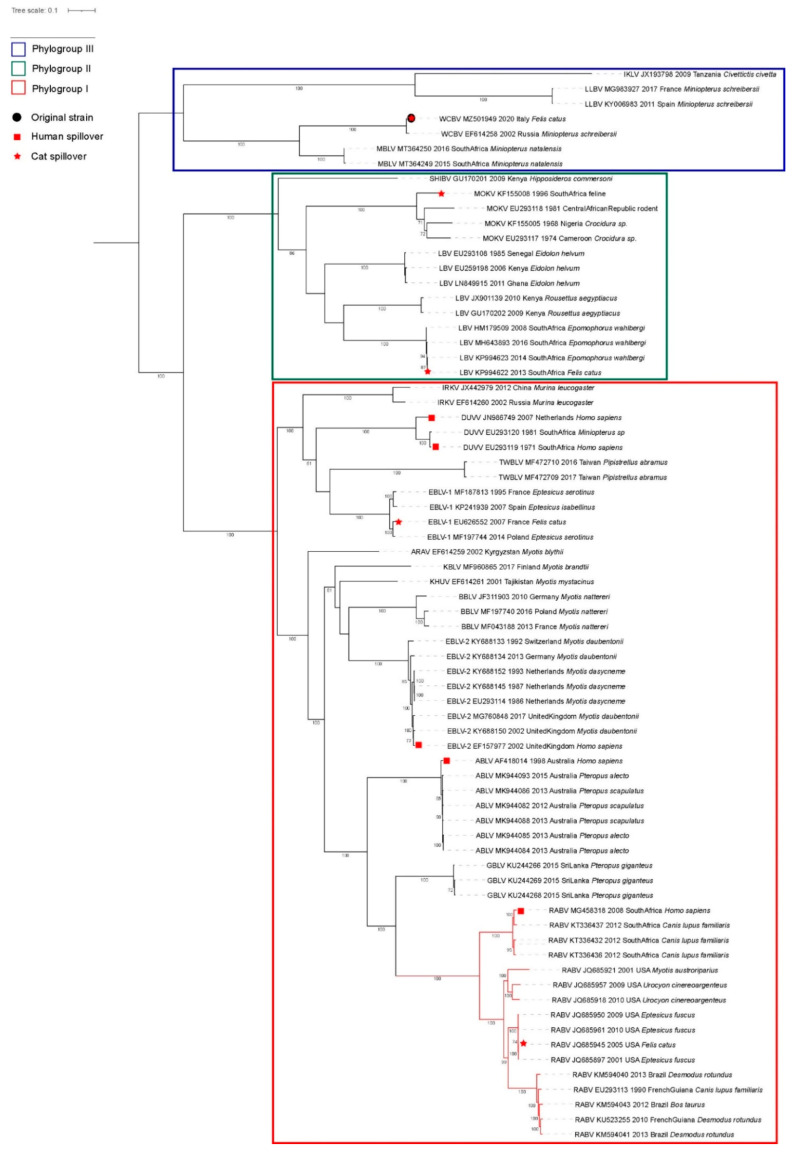
Phylogenetic tree of lyssaviruses. The tree was obtained using PhyML and edited using I-Toll. Bootstrap values > 700 are shown. Within the tree, West Caucasian Bat Lyssavirus is shown as a dashed line, with the original sequence from this study indicated with a black circle. Rabies virus is shown as a red line. In addition, the figure shows some of the reported spillover events of lyssaviruses to humans (red squares) and cats (red stars).

The infectious agent was characterized as WCBV using molecular approaches. We obtained the complete genome of WCBV_Arezzo_Italy_cat_2020 using 43 million reads through a metagenomics approach implemented on the Illumina sequencing platform (Albany, NY, USA). The sequence was deposited in Genbank under accession number MZ501949. Genetic and phylogenetic analyses confirmed our isolate as monophyletic with the reference strain of WCBV (Figure 1), showing 98.7% identity across the whole genome, ranging from 98.5% and 99% over the five genes. We identified only a few point mutations, particularly in the L gene that codes for the RNA dependent RNA polymerase (Table S1).

### 3.2. Tracing of Contacts and Secondary Cases

Epidemiological analyses identified six exposed people among the veterinarians and the owners, who received full PEP. A cohabitant dog and a cat with three kittens were also considered as potentially exposed and were put in quarantine in a public shelter under medical surveillance. All contacts remained healthy as of October 2021, 16 months after the potential exposure. All pets were returned to their home after six months and received further periodic health checks during the entire year.

In the six months following the case, local authorities were frequently contacted by citizen of Arezzo, most often to notify neurological signs either in their pets or in stray animals, or to report biting incidents from animals with compatible signs (Table 1). Active surveillance in the 13 colonies of stray cats that were identified within one (*n* = 2) or three (*n* = 11) kilometres from the index case led to a total of 113 inspections and 82 interviews with the personnel responsible for animal care. Veterinary inspections identified three suspect cases, more specifically one dog and two cats that turned out to be negative. No secondary cases were detected by the passive surveillance on biting animals, as all tested samples were negative for lyssavirus infection (Table 1).

To mitigate the risk of further exposure to positive bats, the access of the tunnel and the riverbed was restricted. On the other hand, authorities decided to install no fences to discourage entrance of cats due to the high risk of flooding.

### 3.3. Spillover Investigation

The cat had no history of travelling abroad so that we assumed she acquired the infection locally, most likely from an infected bat. Passive surveillance implemented in these animals was not effective in tracing the source of infection. Bats submitted to the laboratory mostly belonged to synanthropic species, namely 10 *Pipistrellus kuhlii* and 6 *Hypsugo savii,* as identified using COI. We also analysed a single bent-wing bat that was negative.

**Figure 2:**
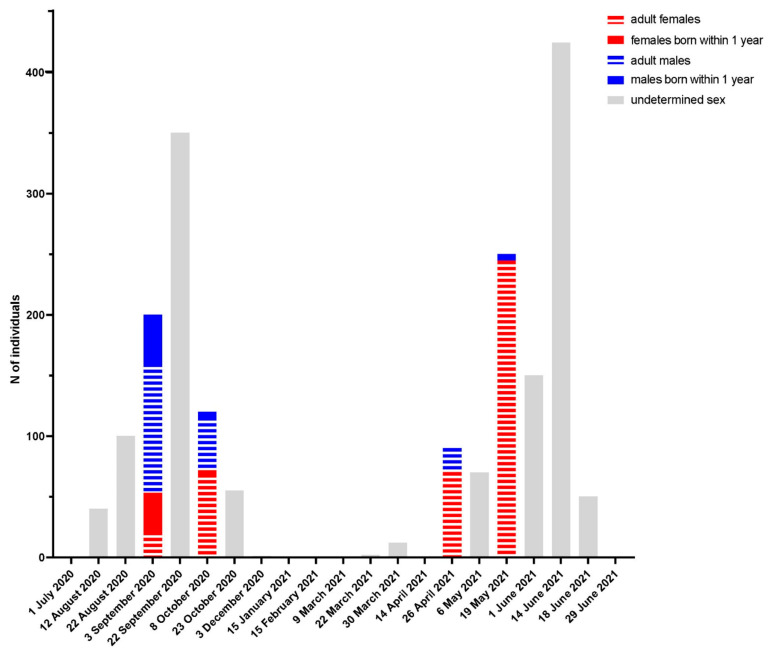
Population trend of *Miniopterus schreibersii* within the tunnel of the underground river. Population size was determined through direct counting, using photographs for larger groups. Each date in the graph represents an actual inspection of the colony. The sex and physiological status were determined only during capture campaigns and has been inferred for the whole population based on the percentage of captured animals and the estimate of the whole population.

We performed an ecological investigation to detect a possible interface between the cat and the bats and to unravel the source and triggers of the spillover. We focused the study on the most likely home-range of the cat according to its sex, age, physiological status and habits, that was calculated as an area of 16.9 hectares surrounded by three highly trafficked roads and the river. We identified several structures across that neighbourhood that were suitable to bats of different species. These included gutters, which are known to frequently host species of the genus *Pipistrellus, Eptesicus* and *Hypsugo* within towns, a ruin and the tunnel of the underground river. This last site was visited in July and again in August, when we spotted a group of Schreibers’ bent-winged bats located a few hundred meters within each entrance. Subsequent bimonthly inspections confirmed that the group occupied the site until November 2020, increasing from around 40 individuals to 350 in late September to leave in November, as shown in Figure 2. Identification of animals during capture campaigns and observations on the aggregation pattern of individuals suggest that males arrive first, followed by adult females. The presence of dark epididymis also suggests mating could occur in this roost in October. The group of bent-winged bats came back to the site in late March 2021, growing to 425 individuals in mid-June before leaving about 15 days later. Differently from the autumn, animals were mostly aggregated in a single large group only on the index entrance.

**Figure 3:**
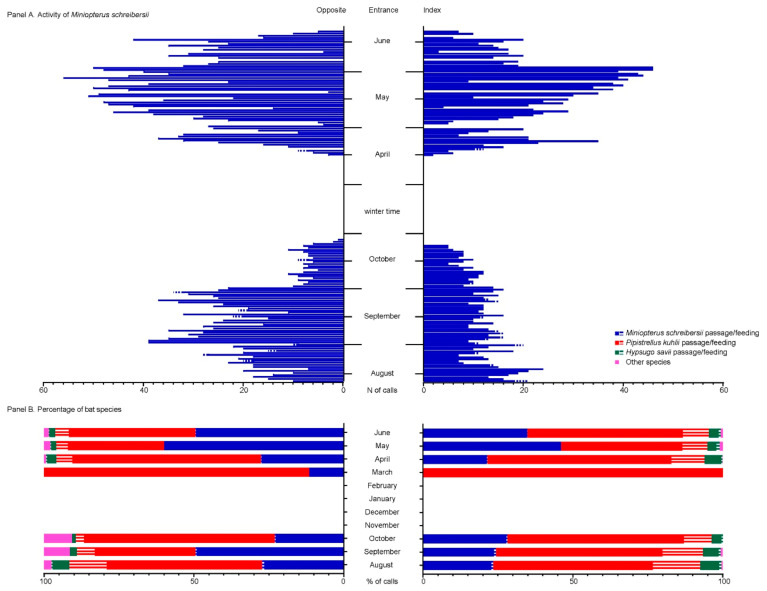
Results from bio-acoustic investigations performed from 12 August to 23 October 2020 and again from 9 March to 29 June 2021. (**A**): temporal trend for the number of bat calls of the bent-winged bat recorded in the two entrances. (**B**): temporal trend in the percentage of calls referred to different species at the two entrances, including the identification of feeding buzzes, compatible with feeding activities.

We confirmed the population trend of bent-winged bats seen during captures using bio-acoustic analyses, that showed a peak between September–October 2020 in the number of echolocation calls recorded from this species and the highest detection of social calls (Figure 3, panel A). No recordings were obtained between November to late March 2021, when echolocation calls increased leading to a new peak in June, that was comparable to data recorded in late summer. Despite the difference seen in the distribution of animals within the tunnel between summer/autumn and spring, bioacoustics supported that bats use both entrances to enter and exit the roost in both seasons (Figure 3, panel A). These analyses supported very low foraging activity for the bent-winged bat on both sides and in both seasons. In detail, mean buzz-ratio from each side was respectively of 1.6% and 1.2% in the index and opposite side over the 72 days of recording in summer-autumn, reduced to 0.3% on both sides in spring; throughout the study period, the majority of days accounted for no feeding calls at all (Figure 3, panel A). On the other hand, data show that the most common species was often the highly synanthropic *Hypsugo savii* that, based on the frequency of feeding buzzes and social calls, uses both areas as a habitual foraging ground (Figure 3, panel B). For comparison, mean feeding activity of this species was respectively 19.5% and 14% at the index and opposite side in summer-autumn, reducing to 14.3% and 9.7% in spring. In addition, we recorded other species, among which *Pipistrellus kuhlii*, frequent on both sides throughout the season, while the finding of other species was sporadic, including *Eptesicus serotinus*, *Hypsugo savii*, *Myotis nattereri*, *Pipistrellus*, *Nyctalus leisleri*, *Myotis emarginatus*, *Myotis myotis/blythii* and *Plecotus* spp. Total species richness was seven in the index site and ten on the opposite side.

**Figure 4:**
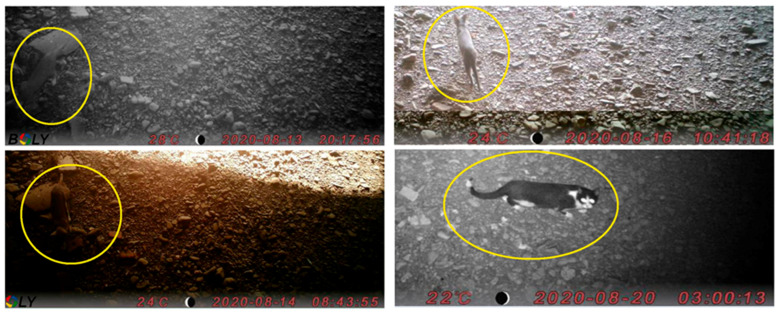
Examples of pictures taken with the camera-trap, showing cats entering and exiting the tunnel in the early morning, daylight, evening and throughout the night.

Camera-traps installed in summer when the river was dry provided significant data during a period of 40 days between mid-August and late September 2020, after which the clarity of the pictures was hampered by the high level of humidity. During this time, we photographed several cats entering and exiting the tunnel from the index entrance, confirming at least one passage in 18 out of 40 days (45%) (Figure 4). Among the other species, we also photographed a hedgehog (*Erinaceus europaeus*), a few non-identified birds and three people.

To investigate the circulation of WCBV in the site, we performed four sampling campaigns in the bat group roosting in the tunnel, obtaining a total of 190 blood samples and 268 oral swabs. In particular, according to the sampling size and the population size, we were able to exclude a minimum of 2% prevalence of viral shedding in October 2020 (Table 2). All salivary samples were negative for the presence of WCBV using the new specific protocol developed in house, that allowed us to increase the analytical sensitivity from 100 (LN34) to 1 RNA copies/µL in salivary samples (Table S2). In addition, results for the LN34 confirmed that all the samples also tested negative for other lyssaviruses.

On the other hand, this investigation was able to confirm the circulation of WCBV through the detection of virus-neutralizing antibodies. The percentage of sero-positive individuals decreased from 42.8% to 10.9% from September 2020 to April 2021, with a new increase to 30.5% seen in May (Table 2). Statistical analyses supported that the month of sampling influenced the outcome of serological analyses, supporting a seasonal variation in the percentage of positivity (*p*-value 0.0057). In autumn, when both sexes from different age classes were sampled, we detected no statistical difference that could be ascribable to the age or sex of the animals. On the other hand, the majority of individuals captured in spring were adult females. Reference sera against EBLV-1, EBLV-2 and RABV were always negative and none of the 30 tested samples from Arezzo’s bent-winged bats cross-reacted against either EBLV-1 or LLEBV.

## 4. Discussion

This report describes the identification of WCBV in a non-flying mammal in the EU. The virus was found in central Italy in a domestic cat showing neurological signs and aggressive behaviour, supporting the ability of WCBV to cause rabies, as is observed for the whole genus *Lyssavirus*. The infection was diagnosed using both DFA and three molecular techniques [39,40,48], which confirms the broad spectrum of diagnostic protocols currently available.

This finding occurred over 2000 km away from, and 18 years after, the discovery of WCBV in the mountains of Caucasus. Nevertheless, the Italian isolate retains a very high identity with the reference strain across the whole genome, showing no signs for geographical clustering or adaptation to a new host. This is peculiar, as most lyssaviruses show a spatio-temporal separation and compartmentalization of diversity based on the host species, that is most notable on the phylogeny of different strains of *Rabies Lyssavirus* [11], shown in Figure 1. Overall, these data are consistent with the direct spillover of WCBV from a broadly distributed and highly vagile natural host rather than the establishment of a new cycle following host-shift. Indeed, WCBV was previously reported from a *Miniopterus schreibersii*, a bat that has long been claimed as the mammal species with the widest distribution on earth [51]. Although the implementation of molecular techniques has led to classification of *M. schreibersii* as a complex rather than a single species, the geographical range of *Miniopterus schreibersii* still spans from Europe to western Transcaucasia [51]. This bat is known to migrate from an average of dozens to fifty kilometres, even if its longest record accounts for 833 km between Spain and France [52,53]. This evidence, paired with the absence of genetic structuring among geographical regions, supports a strong connection between different populations of this species that can facilitate pathogen dispersal across its geographical range [54]. While current knowledge does not support the migration of individual bent-winged bats from Russia to Italy, recent evidence obtained by using hydrogen isotopes revealed that the Tuscany population is more likely to migrate long-distance compared to most European sites. This result is consistent with the lack of geographical clustering of Italian and Russian isolates of WCBV, should this animal be the reservoir [53]. The role of bent-winged bats as natural hosts for lyssaviruses belonging to phylogroup III is supported by the identification of LLEBV in the same *M. schreibersii* and, more recently, by the discovery of MBLV in the South African population of *Miniopterus natalensis* [3].

The hypothesis of the cat acquiring the infection from a bent-winged bat in the city of Arezzo was weakened by the fact that this species was not expected to either roost or feed in urban settings. Indeed, the common bent-winged bat is known to roost almost exclusively underground, especially in caves and abandoned mines [52,55], and to forage in open areas, particularly over freshwaters [56]. Strikingly, our ecological investigation determined how the tunnel that covers the small city river, which was built in the 1950s, actually provides a suitable underground environment for this species in the heart of an urban setting, with up to 425 *Miniopterus schreibersii* recorded intermittently from late March to November 2020. In addition, the active surveillance allowed us to detect neutralizing antibodies against WCBV over four different occasions throughout the different seasons and years (2020 and 2021) and in almost one third of the individuals sampled. We acknowledge that serology alone should be interpreted with caution; however, the fact that target sera failed to neutralize both EBLV-1 and the most closely related LLEBV, which is also associated with the common bent-winged bat in Europe, supports a specific reaction. In addition, we confirmed that reference sera against EBLV-1, EBLV-2 and RABV are not able to neutralize WCBV using the RFFIT test [57,58,59]. No virus was detected in these animals. Based on sampling size and the performances of the test, these data confirm that shedding either involves less than 2% of individuals or is below 1 RNA copies/µL. Overall, we believe that these results provide solid evidence that the cat was infected from a bent-winged bat within the tunnel of the underground river, further strengthening the hypothesis of this bat species as the reservoir for WCBV. In this context, we obtained photographic evidence of cats entering and exiting the tunnel from the south-eastern side, which is located a few hundred meters from the infected bat group and just across a small street from the cat’s house.

Bio-acoustic analyses showed that bats use both entrances throughout the season, suggesting that cats have similar chances to come across bats flying out from both sides of the tunnel. In this context, bio-acoustic analyses confirmed that bats leave the site immediately after they exit to feed. This behaviour is consistent with the current knowledge for this species [56] and should contribute to reduce the interactions between bats and other potential hosts. As a critical point, the bent-winged bat flies very fast, at around 50–70 km/h, which should make it a difficult prey for a cat [55]. However, it is not possible to exclude that the predation happened during the day within the tunnel and not while the animals were leaving their site. In this context, it is crucial to notice that occupancy of the roost varies according to the season, with bats aggregating in a single large group close to the index entrance in spring while stretching out on both sides of the tunnel from August until November. In addition, it is reasonable that clinical signs in the infected bat, including as a minimum an alteration in its ability to fly until grounding and paralysis of the animal, might have facilitated the spillover in this particular setting, even if WCBV was previously reported from an apparently healthy individual [19].

Unfortunately, our data do not provide evidence to restrict exposure to a particular time of the year. Of note, the incubation period of this particular lyssavirus is still unknown and might be longer than expected for RABV, as shown by the death of a woman from the infection with Australian Bat Lyssavirus, 27 months after being bitten from a flying fox [60]. Indeed, further characterization of WCBV’s pathogenicity would provide important data to implement both surveillance and control measures. However, chances for virus transmission are restricted to the seasonal presence of bats within the tunnel. In particular, based on data collected between 2020 and 2021, we determined that the females of the common bent-winged bat occupy this site between late March and the end of June, when they leave the site to give birth elsewhere. Females come back to Arezzo in August, following a group of males that precedes them by about one-two weeks. The whole group formed by females and males stays until November, when it leaves the site to hibernate elsewhere. Seasonal movements of bent-winged bats have been largely described, especially in the Mediterranean countries highlighting a certain variability according to the location and the year of observation, likely as a consequence of local features and yearly environmental phenomena [61,62]. In general, the use of transient roosts while migrating between hibernation and maternity colonies, such as the one identified in Arezzo, is frequent in the species, because roosting requirements follow seasonal patterns [61]. In our case, the females left the site very late during pregnancy, meaning that the nursery or nurseries are located in the proximity of the area. Unfortunately, roost search was beyond the aims of the study. As noted, it is not possible to exclude that external conditions such as minimum temperature and level of rain affect the behaviour of this population over the years.

No virus was found in the bat carcasses screened from Tuscany in summer 2020, leaving the bent-winged bat as the sole species suspected as reservoir host for WCBV in the area. However, passive surveillance in these animals was limited both in terms of diversity of the sampled species and number of animals screened in total; in addition, we cannot rule out the assumption that bats other than *M. schreibersii* may be involved in the ecology of WCBV. To identify secondary cases and investigate the spread of WCBV in other mammals, we enhanced passive surveillance in wild and domestic carnivores from the area. Indeed, the screening of carcasses from suspect cases is largely accepted as the most cost-benefit approach to investigate the occurrence of rabies (RABV) [63]. On the other hand, both pathogenesis and the best approach for surveillance are still debated in the case of other lyssaviruses in bats [64] and therefore we opted for a wide screening of all the bat carcasses submitted to the laboratory regardless of the presence of neurological signs.

Fortunately, no secondary cases were found in carnivores and all animal and human contacts have remained healthy up to now, suggesting the spillover was indeed a stochastic event and the cat was likely not able to pass on the infection. These results are in agreement with field and experimental data that demonstrate how most cross-species transmissions of non-RABV lyssaviruses lead to dead-end infections, with bat variants of RABV rarely causing small outbreaks in other mammals [11,15]. In our specific case, things turned out for the best considering that the efficacy of the vaccine is considered low against the infection with WCBV [20]. As a note, the people exposed to the positive cat received a full PEP anyway. Indeed, the World Health Organization (WHO) encourages the use of PEP to alleviate the psychological burden of fear of rabies in animal-bite victims [1].

Overall, we believe that the spillover of WCBV has several implications that supersede the reality in Arezzo. First, our data support that WCBV is circulating in the Italian bent-winged bat populations, showing no geographical differentiation from the original strain found in Caucasus. In this context, we suggest the possibility that this highly lethal agent is widespread across the whole range of the species, meaning that most European countries are also involved. Considering that another lyssavirus, namely *Lleida bat virus*, was found in this host species in Spain and France [18,65], it would be important to investigate whether these viruses coexist or if they have different ecological niches and, should this be the case, if they relate to distinct populations of *Miniopterus schreibersii*.

Another important finding stemming from our study is the ability of this bat species to adapt to man-made structures even within towns. As underground rivers are quite frequent in Italian towns, we can assume that the presence of this species in urban areas is more common than expected, which raises concerns in terms of public health given that these bats host zoonotic pathogens such as WCBV and LLEBV. In addition, a filovirus, namely *Lloviu virus*, was described from this same host in Hungary and Spain [66,67,68]. Even if the zoonotic potential of this virus is yet to be determined, the phylogenetic relationship with Ebolavirus and Marburgvirus warrants caution [69].

The spillover of WCBV from a bat to a domestic cat and the following exposure of people highlights the possible hazard related to the predatory behaviour of these pets. Besides the obvious consequences for the conservation of wildlife, such a conduct could favour the spillover of zoonotic pathogens, in this case from bats. As a general comment, cats are known to be effective in the predation of bats, and injures from these pets are the first cause of admission in Italian wildlife rescue centres, including the reporting of few bent-winged bats [70]. In this particular case, the deliberate access of the tunnel was restricted, but no actions were undertaken to avoid entrance of free-roaming animals. However, it would be important to investigate possible solutions for upcoming years that could discourage the entrance of cats, while securing minimal disturbance of the colony. Indeed, the bent winged bat is included in the II annex of the Habitats Directive 92/43/CEE and core sites must be managed in accordance with their ecological needs of the species [71].

Furthermore, this case challenges the efficacy of current surveillance programs intended to detect spillover events of bat lyssaviruses, whose description is extremely rare despite the high frequency and spread of different viral species in bats and their supposed ability to infect mammals [16,72,73]. Based on European regulations, Italy is classified free of rabies alongside most EU countries so that the screening of domestic animals is limited if the animal has no travelling history. Indeed, this case was luckily diagnosed thanks to the national laws that require the exclusion of lyssavirus infection in animals displaying neurological signs in absence of an etiological diagnosis [28], but would have been likely to have been missed if no bites had been reported. Indeed, syndromic surveillance is not homogeneously implemented in Europe. Pets showing neurological signs should be screened for rabies regardless of the species and their epidemiological background. This approach has been effective not only to identify several cases of cats infected with EBLV-1 in France [16], but also to highlight secondary RABV infections of dogs when the imported index case was missed [74].

Finally, the case highlights the lack of tools against divergent lyssaviruses and, in general, of accepted national and international guidelines to manage this lethal disease in the absence of an effective vaccine. Indeed, the lack of preventive measures has severe consequences in terms of public health, public opinion and economy. Most importantly, six people were left untreated under medical surveillance, with unknown chances to develop a disease known to have 100% lethality. In addition, the exposed pets had to be kept under quarantine for the whole six-month period, without chances to be shortened upon vaccination, with high expenses, a huge workforce required and a strong emotional impact for the owners. Most activities involving the handling of bats were also suspended, and in the long run this could impact on the conservation of such endangered species. Surely, the spillover case of WCBV has highlighted the importance for the EU at least of developing novel vaccines able to stimulate a pan-lyssavirus neutralising immune response; these should be used to protect domestic animals, with particular reference to predators such as cats, and people more at risk of infection, such as laboratory personnel involved in the diagnosis of rabies or deliberately working with these pathogens, veterinarians, individuals employed in the sector of animal care or exposed to wildlife, such as ecologists, biologists and wildlife rehabilitators [22] whose work normally involves the handling of bats.

## Figures and Tables

**Table 1:** Investigations performed on domestic animals in the city of Arezzo between June and December 2020.

		Dogs	Cats
Notifications of suspect cases		32	14
Notifications of biting animals		23	11
Notifications of free roaming animals		9	3
Active inspection of strayed populations within 1 km from the index case			35
Active inspection of strayed populations 1–3 km from the index case			78
Interview with volunteers working with stray animals	within 1 km		43
	1–3 km away		39
Virological analyses on suspect cases		1	2
Passive surveillance on biting animals (tested for lyssavirus infection)			4

**Table 2:** Biological samples from bent-winged bats analysed in the context of active surveillance.

Date	Salivary Swabs (*n*)	Positive	Prevalence Excluded (%)	Sera (*n*)	Positive	% Positivity
September 2020	75	0	3	56	24	42.8
October 2020	82	0	2	52	16	30.8
April 2021	64	0	2.5	46	5	10.9
May 2021	47	0	7	36	11	30.5

## Data Availability

Meta-genomic data generated in the study were deposited in the Sequence Read Archive (SRA) under the id number SRR13731458. The consensus sequence of WCBV was deposited in Genbank under accession number MZ501949.

## Supplementary Materials

The following are available online at www.mdpi.com/article/10.3390/v13102064/s1, Supplementary methods: Comparison of RT-PCR protocols, Figure S1: Complementarity between WCBV and tested primers and probe, Table S1: Genetic comparison between isolates of WCBV (Caucasus-bat-2002, AN: EF614258 and Arezzo-Italy-cat-2020, AN: MZ501949), Table S2: WCBV analytical sensitivity of two rRT-PCR assays and one end-point RT-PCR in salivary swabs.
