# Supplementary material for: Spillover of West Caucasian Bat Lyssavirus (WCBV) in a Domestic Cat and Westward Expansion in the Palearctic Region
Source: Viruses. 2021 Oct 14;13(10):2064. doi: 10.3390/v13102064 (PMC8540014; doi:10.3390/v13102064)
Supplement: Supplementary file 1 [file viruses-13-02064-s001.zip › viruses-1356584-supplementary.pdf]

Supplemental material to
Spillover of West Caucasian Bat Lyssavirus (WCBV) in a Domestic Cat and Westward Expansion in the Palearctic Region

Supplementary methods

To investigate the sensitivity for WCBV of the rRT-PCR assays used in the study we produced a batch of in vitro transcribed RNA of the target virus. Briefly, amplification of whole 3′UTR and N gene (1683 bp) was carried out on cDNA using the Platinum™ Taq DNA Polymerase (Invitrogen) (primer set available upon request). The PCR amplicon was purified from 0.8% agarose gel by NucleoSpin® Gel and PCR Clean-up kit (Macherey-Nagel), cloned with TA Cloning Kit Dual Promoter (Invitrogen) into vector pCRII (Invitrogen) and transformed into DH5α Competent Cells (ThermoFisher Scientific) according to the manufacturer's instructions. We then identified bacterial colonies inserted with the WCBV sequence fragment through PCR, using primers M13 (5′-GTAAAACGACGGCCAG-3 and 5′-CAGGAAACAGCTATGAC-3′) and Platinum™ Taq DNA Polymerase (Invitrogen). Selection of positive bacterial colonies was carried out through PCR using primers M13 FW and REV targeting vector sequence; selected bacterial colonies were further grown. Plasmid DNA was extracted using a commercial kit (GenElute™ Plasmid Miniprep Kit, Merck) according to the manufacturer's instructions. PCR was carried out using Platinum™ Taq DNA Polymerase (Invitrogen) and primers M13 FW and REV. Transcription was performed using MEGAscript™ T7 Transcription Kit (Invitrogen) following the manufacturer's instructions and in vitro transcribed RNA was purified using MEGAclear™ Transcription Clean-Up Kit (Invitrogen) following the manufacturer's instructions. To control the quality of the process we performed a one-step RT-PCR followed by Sanger sequencing [1], and we measured the concentration of WCBV RNA first using NanoDrop™ Lite Spectrophotometer (Thermo Fisher Scientific) and then Qubit 4 Fluorometer (Invitrogen). We calculated the number of RNA copies and diluted the RNA in nuclease free water to the concentration 1.00E+07 RNA copies/µl. The ten-fold serial dilutions were prepared up to 1 RNA copies/µl in a pool of lyssavirus-negative RNA extracted from pool of oral swabs from miniopterus bats (*Miniopterus schreibersii*). Host RNA was extracted by NucleoSpin RNA kit (Macherey-Nagel) according to the manufacturer's instructions. The analytical sensitivity for WCBV in oral swabs of bats was tested using three rRT-PCR and one end-point RT-PCR as described elsewhere [1–3], including a modified rRT-PCR protocol that was developed during this study using the LN34 probe [3] with modified primers that enhanced the sensitivity of the assay for WCBV. Complementarity between WCBV and oligonucleotides is presented in figure S1. Real-time PCRs were carried out on CFX96 Touch Deep Well Real-Time PCR System (Biorad) and analysed by CFX Maestro qPCR Analysis Software. Results are presented in tables S2.

Figure S1. Complementarity between WCBV and tested primers and probe. WCBV_Arezzo_Italy_cat_2020 is selected as reference strain and highlighted in yellow. Degenerations in oligonucleotides are highlighted in grey.

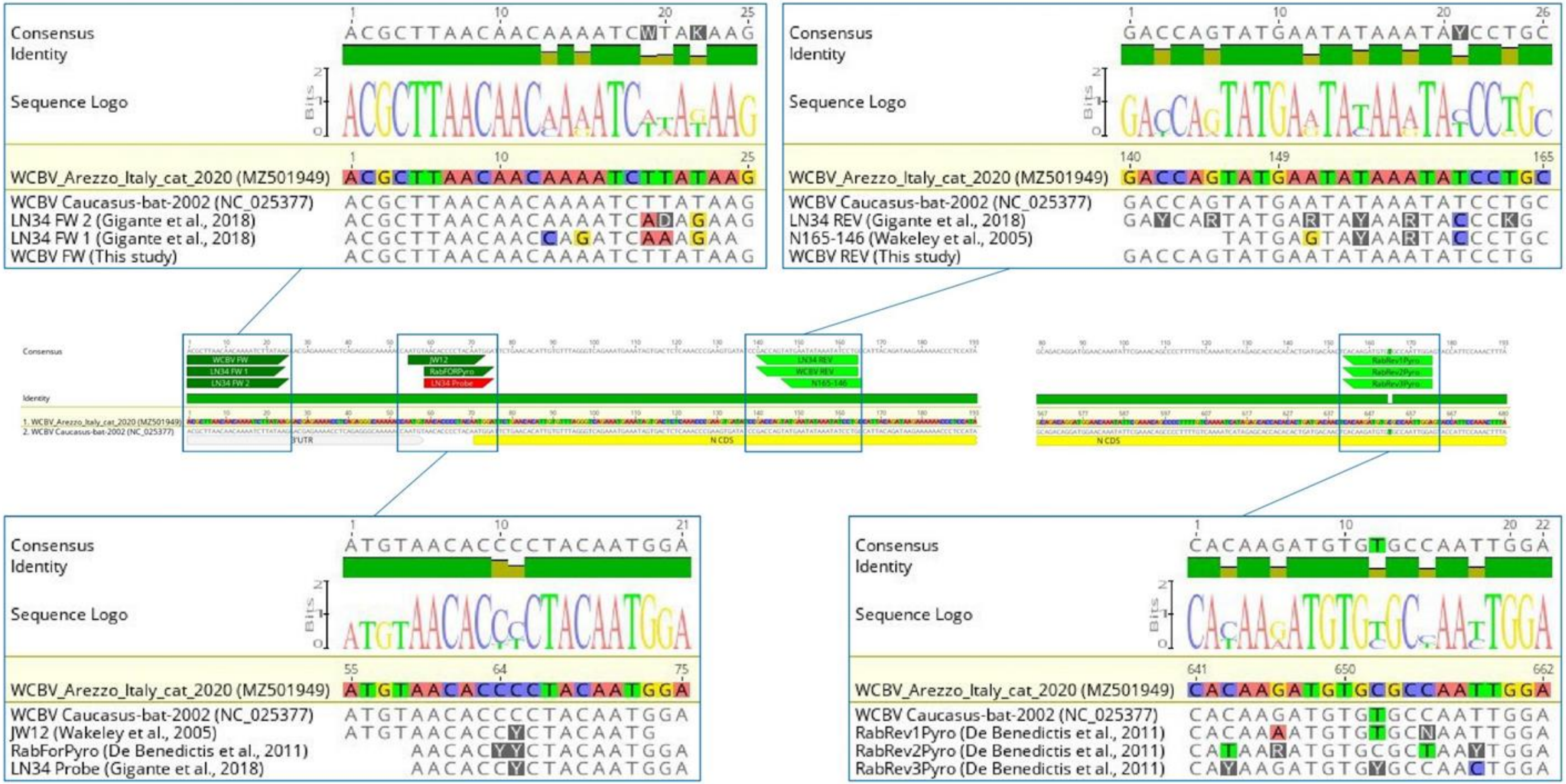

Table S1: Genetic comparison between isolates of WCBV (Caucasus-bat-2002, AN: EF614258 and Arezzo-Italy-cat-2020, AN: MZ501949)

Protein	Length	Nucleotidic similarity (%)	Number of mutations	
			nucleotidic	amminoacidic
N	1353 nt (451 aa)	99.0	14	0
P	894 nt (298 aa)	98.5	13	3
M	609 nt (203 aa)	99.0	6	1
G	1578 nt (526 aa)	98.8	19	1
L	6384 nt (2128 aa)	98.9	68	7
Complete genome	12278 nt	98.7	157	12

Table S2. WCBV analytical sensitivity of two rRT-PCR assays and one end-point RT-PCR. *In vitro* transcribed WCBV RNA was diluted in a pool of RNA extracted from oral swabs of bats. C_t values are reported as the mean of three replicates ± standard deviation. The limit of detection (LoD) is the highest dilution at which all the replicates test positive (highlighted in grey). For rRT-PCR, the diagnostic cut-off was set as a C_t value ≤ 35. Only values below the diagnostic cut-off are considered positive. * At least one replicate gave negative results.

RNA copies/µl	rRT-PCR WCBV specific (this study)	rRT-PCR LN34[3]	End-point RT-PCR[1]
**10000**	21.2 ± 0.09	25.6 ± 0.35	Positive
**1000**	24.7 ± 0.06	29.1 ± 0.42	Positive
**100**	28.2 ± 0.11	33.2 ± 0.42	Positive
**10**	31.8 ± 0.40	36.6 ± 0.75	Negative*
**1**	35.3 ± 0.18	Negative	Negative

Supplementary references

[1] De Benedictis P, De Battisti C, Dacheux L, et al. Lyssavirus detection and typing using pyrosequencing. J. Clin. Microbiol. 2011;49:1932–1938.

[2] Cai X, Chen J, Hu J, et al. A Peptide-based Magnetic Chemiluminescence Enzyme Immunoassay for Serological Diagnosis of Corona Virus Disease 2019 (COVID-19). medRxiv. 2020;2020.02.22.20026617.

[3] Gigante CM, Dettinger L, Powell JW, et al. Multi-site evaluation of the LN34 pan-lyssavirus real-time RT-PCR assay for postmortem rabies diagnostics. PLoS One. 2018;13:1–25.
